# Promoting Glutathione Synthesis: A Possibility for Treating Cardiomyopathy Induced by a Maternal Western Diet

**DOI:** 10.3390/nu16152520

**Published:** 2024-08-01

**Authors:** Jialing Zhang, Jiayu Wang, Da Xu, Yiting Gui, Fan Bai, Yu Huo, Li Cao, Yonghao Gui

**Affiliations:** 1 Institute of Pediatrics, Children’s Hospital of Fudan University, National Children’s Medical Center, Shanghai 201102, China; zhangjl_61@163.com (J.Z.);; 2 National Health Commission (NHC) Key Laboratory of Neonatal Diseases, Fudan University, Shanghai 201102, China; 3 Cardiovascular Center, Children’s Hospital of Fudan University, Shanghai 201102, China; 4 Ultrasound Department, Obstetrics and Gynecology Hospital of Fudan University, Shanghai 200090, China

**Keywords:** cardiomyopathy, developmental origins of disease, glutathione, reactive oxygen species, oxidative phosphorylation

## Abstract

Background: The adverse effects of a Western diet on obesity and diabetes among reproductive-aged women pose a significant threat to the cardiovascular health of their offspring. Given the crucial role of glutathione metabolism and glutathione-related antioxidant defense systems in cardiovascular diseases through scavenging ROS and maintaining redox homeostasis, further exploration of their specific influence is imperative to develop therapeutic strategies for cardiomyopathy induced by a maternal Western diet. Methods: We developed a prenatal maternal Western diet exposure model in C57/B6 mice to investigate cardiac morphology and function through histological analysis and echocardiography. RNA sequencing and analysis were utilized to elucidate the mechanisms underlying the impact of a maternal Western diet and N-acetylcysteine treatment on cardiomyopathy. Additionally, ELISAs, transmission electron microscopy, and flow cytometry were employed to assess the antioxidant defense system and mitochondrial ROS levels in progenitor cardiomyocytes. Results: N-acetylcysteine significantly mitigated cardiomyocyte hypertrophy, myocardial interstitial fibrosis, collagen type I accumulation, and left ventricular remodeling induced by a maternal Western diet, particularly in male offspring. Furthermore, N-acetylcysteine reversed the increase in apoptosis and the increase in the β/α-MyHC ratio in the myocardium of offspring that results from a maternal Western diet. RNA sequencing and GSEA revealed that the beneficial effects of N-acetylcysteine were linked to its ability to modulate oxidative phosphorylation pathways. Additionally, N-acetylcysteine treatment during pregnancy can markedly elevate glutathione levels, augment glutathione peroxidase (GPx) activity, and mitigate the accumulation of mitochondrial ROS caused by a maternal Western diet. Conclusions: N-acetylcysteine mitigated cardiomyopathy induced by a maternal Western diet by bolstering glutathione synthesis and enhancing GPx activity, thereby scavenging mitochondrial ROS and modulating oxidative phosphorylation pathways.

## 1. Introduction

The alarming increase in obesity and type 2 diabetes among women of reproductive age is a growing concern [1], with the Western diet serving as a significant contributor [2]. This dietary pattern not only poses a threat to the mother’s health but also carries significant risks for the health of her offspring. The developing cardiovascular system in utero is highly susceptible to environmental insults, including the nutritional components of mothers [3,4]. Some studies have shown that a maternal Western diet results in cardiometabolic dysfunction, including hypertension and hyperglycemia, in offspring [5]. However, the understanding of the specific impacts of a maternal Western diet on the structure and function of the heart in offspring is limited.

Our previous studies showed that maternal obesity induced by a Western diet results in heart weight abnormalities, left ventricular remodeling, vasculitis, and an inflammatory response in offspring [6,7,8,9]. However, the specific impact of a maternal Western diet on the metabolism and function of cardiomyocytes and the extracellular matrix viscoelasticity in offspring remains unexplored and requires further elucidation.

Mitochondria produce the majority of reactive oxygen species (ROS) during oxidative phosphorylation (OXPHOS) and ATP generation. If not properly controlled, ROS can cause oxidative damage to tissues and cells, initiating a harmful cycle of inflammation and escalating oxidative stress, despite their pivotal role in maintaining healthy cellular and mitochondrial signaling and functionality [10]. Recent studies have investigated mitochondrial dysfunction and oxidative stress as key mechanisms involved in the development of cardiomyopathy in diabetes and/or obesity [11,12]. Mitochondrial ATP production sustains the energetic demands of cardiomyocytes, whereas mitochondrial dysfunction can exacerbate or initiate cardiomyopathy by promoting oxidative stress, metabolic reprogramming, dysregulated intracellular signaling, and mitochondrial apoptosis in cardiomyocytes. Obesity and hyperglycemia, generally induced by a Western diet, are directly linked to mitochondrial activity, function, and oxidative stress [13]. The dysregulation of mitochondrial homeostasis leads to the excessive production of mitochondrial reactive oxygen species (mtROS), which causes oxidative damage to mitochondrial DNA (mtDNA) and the release of mtROS into the cytoplasm. mtROS mediate cardiomyocyte pyroptosis and microvascular dysfunction [14,15], thereby playing an active role in the pathogenesis of cardiomyopathy.

When confronted with diverse pathological challenges, the mammalian heart, characterized by abundant mitochondria and substantial oxygen requirements, becomes vulnerable to oxidant generation and subsequent oxidative damage. The glutathione (GSH) system is one of the most potent endogenous antioxidant systems in the cardiovascular system owing to its pivotal role in scavenging excessive ROS and maintaining redox homeostasis [16]. Hence, the targeted enhancement of the GSH system in cardiomyopathy patients holds promise as a therapeutic strategy.

N-acetylcysteine, the acetylated derivative of the amino acid L-cysteine, serves as a thiol group (SH) source and can promote GSH biosynthesis [17]. N-acetylcysteine has been employed as a mucolytic agent and an antidote to acetaminophen poisoning for several decades. Recently, with the enhanced understanding of the mechanisms of action of N-acetylcysteine, its clinical applications have been broadened. The therapeutic administration of N-acetylcysteine has proven to be beneficial for managing oxidative stress and inflammation due to its ability to regulate glutamate homeostasis [18]. Although numerous clinical trials have established the potential of N-acetylcysteine for clinical use in disorders of the central nervous system [19,20,21], its application in maternal Western diet-induced cardiomyopathy remains unexplored.

In this study, we hypothesized that N-acetylcysteine could alleviate oxidative damage and cardiomyopathy caused by a maternal Western diet by promoting GSH synthesis. To validate this hypothesis, we employed a mouse model and conducted experiments to elucidate the underlying mechanisms and identify potential intervention targets.

## 2. Materials and Methods

### 2.1. Animals and Experimental Procedures

The experiments with animals in this study were conducted in strict accordance with the ethical standards outlined by the National Institutes of Health for the Care and Use of Laboratory Animals. The experimental protocol was approved by the Animal Experiment Committee of the Children’s Hospital of Fudan University, with the approval number 328-2019 and an approval date of 25 December 2019. For the humane termination of the animals, carbon dioxide (CO_2_) was used as the euthanasia method.

**Figure 1:**
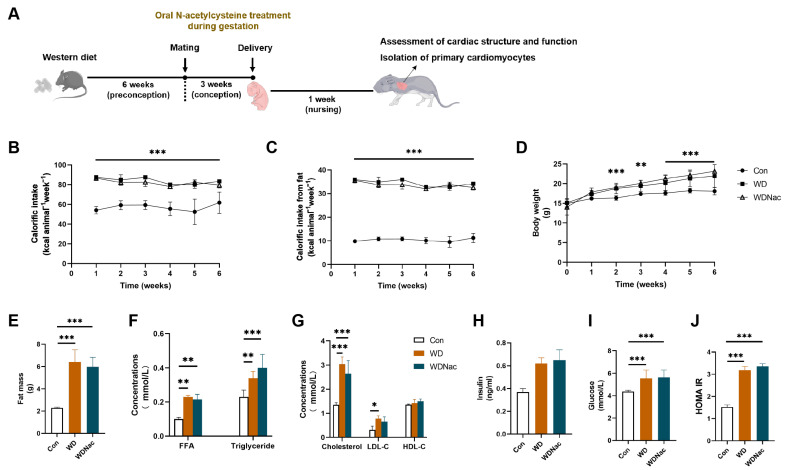
Experimental procedures and characteristics of dams administered a control diet (Con) and a Western diet with (WDNac) or without (WD) N-acetylcysteine treatment during pregnancy. (**A**) Experimental diagram. (**B**–**D**) Calorific intake, calorific intake from fat, and body weight in dams fed either the control or Western diet during 6 weeks’ feeding (*n* = 8). (**E**) Maternal fat mass after 6 week’s different diet feeding (*n* = 5). (**F**–**J**) Maternal fasting serological metabolic analysis after 6 week’s feeding (*n* = 5). FFA, free fatty acid; LDL-C, low-density lipoprotein cholesterol; HDL-C, high-density lipoprotein cholesterol; HOMA-IR, homeostatic model assessment for insulin resistance. *** *p* < 0.001, ** *p* < 0.01, * *p* < 0.05.

An overview of the experimental procedures is depicted in Figure 1A. A total of 24 female and 10 male C57/BL6 mice, aged 3 to 4 weeks, were procured from Shanghai SLACCAS Experimental Animal Ltd. (Shanghai, China). The sample size was calculated using the resource equation approach (K = 3). The mice were allowed to acclimate to the laboratory environment for one week before the experiments commenced. The animals were housed in a controlled environment with alternating 12 h periods of darkness and light. Following the acclimation period, the female mice were randomly assigned to either the control group (Con group) or the WD group. The animals were provided either a control diet (18.2 kcal% fat, P1103F-25, SLACOM, Shanghai, China) or a Western diet (41 kcal% fat, Western Diet D12079B, Research Diets, New Brunswick, NJ, USA), which was supplemented with a 20% sucrose solution (3.9 kcal/g), including vitamins (Vitamin Mix V10001, Research Diets, New Brunswick, NJ, USA) and minerals (Mineral Mix S10001, Research Diets, New Brunswick, NJ, USA), following established protocols. The male mice were fed a standard laboratory diet. The body weights of the animals were monitored regularly, and the fat masses of the dams were measured using a nuclear magnetic resonance (NMR) analyzer (MesoQMR23-060H, NIUMAG, Suzhou, China). Following a dietary intervention spanning six weeks, all the females were mated with males of the corresponding age. Subsequently, the female mice in the WD group were randomly assigned to receive oral N-acetylcysteine treatment (1 g/kg per day, A9165, Sigma-Aldrich, St. Louis, MO, USA; WDNac group) through their drinking water or not (WD group) during gestation. N-acetylcysteine was administered in drinking water at 4–5 mg/mL, periodically adjusted every two days to maintain a calculated daily intake of 1 g/kg body weight for pregnant C57/BL6 mice, considering their average water consumption (approx. 8 mL/day) and weight range (24–40 g).

Newborn mice, either on day 1 or 7 after birth, were anesthetized using isoflurane inhalation (3%). Their length was accurately measured from the tip of the nose to the anus, with the mice positioned prone. Following the measurement, the mice were humanely decapitated. There were no exclusions. The individual sex (male or female) was determined using genotypic sex determination (GSD), as described previously [22]. Additionally, to evaluate the body mass index of the mice, Lee’s index was calculated utilizing the following formula: body weight (g)^1/3^/body length (cm) × 1000. The heart of each mouse was weighed, the cardiac function was assessed, and cardiac morphological examinations were conducted.

### 2.2. Serological Metabolic Analysis

Blood samples were obtained from the tails of dams following a 4 h fast. After allowing the samples to clot for 60 min, the serum was isolated via centrifugation. A Cobas 6000 modular analyzer (Cobas E601 module, Roche, Basel, Switzerland) was utilized to quantitatively determine the concentrations of fasting blood glucose, free fatty acids (FFAs), triglycerides, total cholesterol, low-density lipoprotein cholesterol (LDL-C), and high-density lipoprotein cholesterol (HDL-C). Furthermore, to measure insulin levels in the fasting state, a Mouse Insulin ELISA Kit (90080, Crystal Chem, Elk Grove Village, IL, USA) was used following the manufacturer’s instructions. Ultimately, the insulin resistance index (HOMA-IR) was calculated using the following formula: (fasting plasma glucose [mmol/L] multiplied by fasting serum insulin [mU/L]) divided by 22.5.

### 2.3. Histological Analysis

To gain insight into the histological structure of neonatal mouse hearts, we performed a detailed analysis of excised heart tissues. Immediately following excision, the hearts were submerged in a 4% paraformaldehyde solution for 24 h to achieve optimal fixation. Once fixed, the hearts were embedded in paraffin and then precisely sliced into 5-µm-thick sections.

For hematoxylin and eosin (HE) staining, an assessment of cardiac hypertrophy was conducted utilizing the staining protocol provided by the Hematoxylin and Eosin (HE) staining kit (C0105M, Beyotime Biotechnology Co., Ltd., Shanghai, China). Briefly, the tissue sections underwent deparaffinization and rehydration procedures, followed by staining with hematoxylin solution for 10 min, and subsequently with eosin solution for 2 min. After staining, sections were rehydrated in ethanol, cleared with xylene, and mounted with resin for microscopic examination.

To assess myocardial cell areas, we utilized wheat germ agglutinin (WGA, W21405, Thermo Fisher, Waltham, MA, USA) as a fluorescent marker and measured the areas of individual cardiomyocytes within neonatal mouse hearts. After undergoing standard deparaffinization and rehydration procedures, the sections were incubated with diluted WGA conjugates (5.0 µg/mL) for 30 min at room temperature. Following the incubation, the sections were carefully rinsed and subsequently incubated with DAPI solution (1:1000 dilution, #4083, Cell Signaling Technology, Danvers, MA, USA) for an additional 5 min. Once thoroughly rinsed, the sections were then mounted for further analysis.

For comprehensive histological analysis, the heart sections were subjected to Masson’s trichrome staining using a Masson’s Trichrome Staining Kit (C0189S, Beyotime Biotechnology Co., Ltd., China), After standard deparaffinization and rehydration procedures, the sections were treated with 50 μL of hematoxylin staining solution for 5 min, followed by a thorough rinse. Subsequently, 50 μL of Ponceau S-Acid Fuchsin staining solution was applied for 10 min, after which the sections were quickly rinsed. Then, 50 μL of phosphomolybdic acid differentiation solution was added for 2 min. Afterward, 50 μL of Brilliant Green staining solution was applied for 1 min, and the sections were gently rinsed. Following graded alcohol dehydration, the sections were mounted and prepared for examination. Furthermore, for evaluation purposes, picrosirius red staining was carried out with the utilization of polarized light detection. The sections underwent a graded ethanol series for deparaffinization and rehydration. Subsequently, the sections were incubated in a picrosirius red staining solution (ab246832, Abcam, Boston, MA, USA) for one hour. Following this, the sections were washed, dehydrated, and mounted for examination. The immunohistochemical staining of collagen type I (COL I) and collagen type III (COL III) was also performed using the fixed slices to visualize collagen fibers in the hearts of neonates. Specific primary antibodies were employed, including a Collagen Type I antibody (1:100 dilution, 600-403-103, Thermo Fisher, Waltham, MA, USA) and a Collagen Type III antibody (1:500 dilution, 600-401-105-01, Thermo Fisher, Waltham, MA, USA). Peroxidase rabbit secondary antibodies (1:10,000 dilution) were appropriately utilized. The percentages of fibrosis within the tissue area were determined using Masson’s trichrome staining and histochemistry scores (H-scores).

To evaluate cell apoptosis, proliferation, and cardiomyocyte function in the heart, we conducted immunofluorescence staining, utilizing specific markers. Cell apoptosis in heart tissue sections was assessed using a Terminal deoxynucleotidyl transferase dUTP nick end labeling (TUNEL) Apoptosis Assay Kit (C1089, Beyotime Biotechnology Co., Ltd., China). The tissue sections were dewaxed, rehydrated, and treated with DNase-free proteinase K (20 μg/mL) for 20 min. After thorough washing, the sections were incubated with 50 μL TUNEL solution for one hour at 37 °C, followed by nuclear staining with DAPI solution (1:1000 dilution, #4083, Cell Signaling Technology). The sections were then gently rinsed and mounted for examination. Ki-67 staining was used to quantify cellular proliferation using a Ki67 Cell Proliferation Assay Kit (C2301S, Beyotime Biotechnology Co., Ltd., China). Briefly, paraffin sections are initially subjected to standard dewaxing and hydration procedures. An immunostaining blocking solution is applied and allowed to block for 10 min, then a Ki67 rabbit monoclonal antibody is added, incubating overnight at 4 °C. After thorough washing, an anti-rabbit Cy3 secondary antibody was introduced and incubated for one hour. Subsequently, a nuclear staining DAPI solution (1:1000 dilution, #4083, Cell Signaling Technology) is added, staining the nuclei for 5 min. After further washing, the slides are mounted and observed under a fluorescence microscope. Furthermore, immunofluorescent staining for α-MyHC and β-MyHC was performed on paraffin sections to evaluate the expression of cardiomyocyte-specific proteins. Following dewaxing, hydration, antigen retrieval, and BSA serum blockade, specific primary antibodies were used overnight at 4 °C. These included an α-MyHC antibody (1:100 dilution, A9516, Abclonal, Wuhan, China) and a β-MyHC antibody (1:100 dilution, A4963, Abclonal, Wuhan, China). Subsequently, peroxidase-conjugated rabbit secondary antibodies were incubated for one hour, followed by nuclear staining with DAPI solution (1:1000 dilution, #4083, Cell Signaling Technology). The slides were then washed, mounted, and observed under a fluorescence microscope. The percentages of positive cells detected using immunofluorescence staining and the β-MyHC-to-α-MyHC ratio were subsequently measured, providing a quantitative assessment of cardiomyocyte function in the offspring.

### 2.4. Echocardiographic Evaluation

To precisely determine the left ventricular morphology and function in 7-day-old neonatal mice, echocardiography was performed utilizing a high-resolution imaging system (Vevo 2100, VisualSonics, Toronto, ON, Canada). Prior to the procedure, the pups were anesthetized via isoflurane inhalation, maintaining a concentration between 1% and 1.5%. The left ventricular posterior wall thickness (LVPW), left ventricular internal dimension (LVID), and interventricular septum (IVS) were measured during both the end-systolic and end-diastolic phases of the cardiac cycle. Then, the collected data were subsequently processed using VisualSonics software (version 2.2.0.), which enabled the calculation of the LV mass, left ventricular ejection fraction (LVEF), and left ventricular fraction shortening (LVFS).

### 2.5. RNA Sequencing and Functional Enrichment Analysis

The total RNA was extracted with TRIzol reagent (Invitrogen, San Diego, CA, USA). Subsequently, the purity and concentration of the extracted RNA were rigorously assessed using a NanoDrop 2000 spectrophotometer (Thermo Scientific, Boston, MA, USA). A TruSeq Stranded mRNA LT Sample Prep Kit (Illumina, San Diego, CA, USA) was utilized to prepare the sequencing libraries. Subsequent transcriptome sequencing and comprehensive analysis were performed as described previously [23]. For a comprehensive evaluation, genome-wide transcriptomic profiling was conducted on five independent experimental replicates in males and four in females. Differential expression analysis was conducted using the DESeq (2012) R package, with a significance threshold set at *p* < 0.05 and a fold change > 2 or a fold change < 0.5. Additionally, the Gene Ontology (GO) enrichment analysis of DEGs and gene set enrichment analysis (GSEA) were performed to identify distinct phenotypic differences between groups.

### 2.6. Antioxidant Capacity Assays

Protein was extracted from mouse pup hearts via homogenization with radioimmunoprecipitation assay (RIPA) lysis buffer (C500007, Sangon Biotech, Shanghai, China) and centrifugation at 12,000× *g* for 20 min at 4 °C. Protein concentrations were determined using a BCA protein assay kit (#P0011, Beyotime Biotechnology). To evaluate the antioxidant status of the heart tissues, the glutathione (GSH) concentration was assessed with a reduced glutathione assay kit (A006-1-1, Nanjing Jiancheng Bioengineering Institute, Nanjing, China) according to the manufacturer’s instructions, and the activity of glutathione peroxidase (GPx) was measured with a glutathione peroxidase assay kit (A005-1-2, Nanjing Jiancheng Bioengineering Institute) according to the manufacturer’s instructions. Additionally, the total antioxidant capacity (T-AOC) refers to the comprehensive ability of all antioxidant substances within a biological system to counteract free radicals and oxidative stress. Specifically, it was quantified through employing a total antioxidant capacity assay kit (A015-1-2, Nanjing Jiancheng Bioengineering Institute), according to the manufacturer’s instructions.

### 2.7. ROS Assay

To measure ROS levels in mouse heart tissue, we employed a dihydroethidium (DHE) fluorescent probe (D7008, Sigma, USA). The heart tissue was fixed in 4% paraformaldehyde overnight at 4 °C. It was then dehydrated in 15% and 30% sucrose solutions. The tissue was embedded in OCT, frozen with liquid nitrogen, and stored at −80 °C. Using a cryostat, 10 μm sections were cut and stained with DHE probe solution (1:500 dilution) for 25 min at 37 °C. Next, DAPI solution (1:1000 dilution, #4083, Cell Signaling Technology) was added and incubated for 5 min. After thorough washing, the sections were mounted for examination. Images were obtained using a fluorescence microscope, and the fluorescent-positive areas were analyzed utilizing ImageJ software (version 2.14.0).

### 2.8. Mitochondrial Ultrastructure

The mitochondrial ultrastructure in the myocardial tissue was analyzed using transmission electron microscopy as described previously [24]. Briefly, freshly dissected tissue samples from the left ventricular (preperfused with PBS) were fixed overnight using 2.5% glutaraldehyde, sequentially incubated with 1% osmium for 1 h and immersed in 2% uranyl acetate for 30 min. After dehydration in a gradient of ethanol and acetone, the samples were embedded in Epon resin. Ultrathin sections were contrasted with 1% uranyl acetate and lead citrate. Areas containing mitochondria were randomly selected for each sample and visualized using transmission electron microscopy (TEM) (HT7700, Hitachi, Tokyo, Japan).

### 2.9. Isolation, Purification, and Culture of Primary Cardiomyocytes

Primary cardiomyocytes were isolated from 7-day-old neonatal mice. After the mice were fixed in the supine position and the chest and neck were sterilized with 75% alcohol, the hearts were excised rapidly under aseptic conditions. Residual blood was expelled by gently pressing the heart. The atria and large vessels were trimmed, and the ventricular tissue was minced into small pieces on ice. A mixture of enzymes from a mouse cardiac dissociation kit (130-098-373, Miltenyi Biotec, Bergisch Gladbach, Germany) was prepared, and the tissue fragments were transferred to a C-tube containing the enzyme mixture. Using a tissue homogenizer (gentleMACS™ Octo Dissociator with Heaters, Miltenyi Biotec, Germany), the cells were dissociated into a single-cell suspension at 37 °C (mr_NHDK_1 procedure). The suspension was diluted with warmed culture medium, filtered, and centrifuged to remove the supernatant. Cardiomyocytes were resuspended in mouse cardiomyocyte culture medium (CM-M073, Wuhan Pricella Biotechnology Co., Ltd., Wuhan, China) and cultured for 90 min. Nonadherent cells were transferred to a new plate, and cardiomyocytes were purified through differential adherence.

Cardiomyocytes were then identified using α-Actinin immunofluorescence staining. Cells were fixed using 4% paraformaldehyde for 10–15 min at room temperature. Subsequently, the cells were permeabilized with 0.1% Triton X-100 for 5 min and blocked with 1% BSA in PBS for 30 min. A diluted α-actinin primary antibody (1:25 dilution, #3134, Cell Signaling Technology) was then applied and incubated overnight at 4 °C. After rinsing, a diluted fluorescently labeled secondary antibody (1:1000 dilution, #4413, Cell Signaling Technology) was added and incubated for 1 h. Following this, DAPI solution (1:1000 dilution, #4083, Cell Signaling Technology) was applied for 5 min to stain the cell nuclei. Finally, the expression and localization of α-Actinin in cardiomyocytes were observed using a fluorescence microscope.

### 2.10. MitoSOX Assay

To measure mitochondrial ROS levels in cardiomyocytes, we used a MitoSOX probe (M36008, Thermo). The procedure included diluting the stock solution to 5 µM, incubating the primary cardiomyocytes with the working solution for 30 min at 37 °C, and analyzing the cells with a flow cytometer (Canto, BD, Franklin Lakes, NJ, USA) on the PE channel.

### 2.11. Statistical Analysis

Statistical analysis was conducted using SPSS version 21.0. To assess parametric data, the mean ± standard deviation (SD) was used as the measure of central tendency and dispersion. For comparisons among multiple groups, statistical significance between each group was examined using a one-way or two-way ANOVA followed by Bonferroni correction for multiple analyses. Statistical significance was set at a *p* value of <0.05. Furthermore, the notation “*n*” signifies the count of dams or litters within each group, where each experimental unit comprised a single male or female pup originating from a single litter.

## 3. Results

### 3.1. N-Acetylcysteine Restores Heart Abnormalities in Offspring Caused by a Maternal Western Diet

The total energy intake and energy intake from fat in the three groups of dams during the six-week period of the Western diet feeding are shown. The results indicated that the WD and WDNac groups exhibited significantly greater total energy intake than the control group (Con) did, with the majority of their energy intake stemming from fat (Figure 1B,C). Changes in body weight throughout the six-week period revealed that the WD and WDNac groups exhibited significantly greater body weights than the Con group did from the second week onward (Figure 1D). After six weeks of Western diet feeding, the body compositions of the dams were assessed using NMR analysis. Notably, the WD and WDNac groups exhibited significant increases in fat mass compared to the Con group (Figure 1E). Furthermore, the serological metabolic analysis of the dams demonstrated that the WD and WDNac groups had significantly greater concentrations of free fatty acids (FFAs) and triglycerides than the control group did (Figure 1F). Additionally, the total cholesterol levels in the WD and WDNac groups were significantly elevated, accompanied by a marked increase in low-density lipoprotein cholesterol levels (Figure 1G). Unsurprisingly, the WD and WDNac groups also exhibited significantly higher fasting blood glucose levels with a concomitant increase in HOMA-IR than those in the control group (Figure 1H–J).

**Figure 2:**
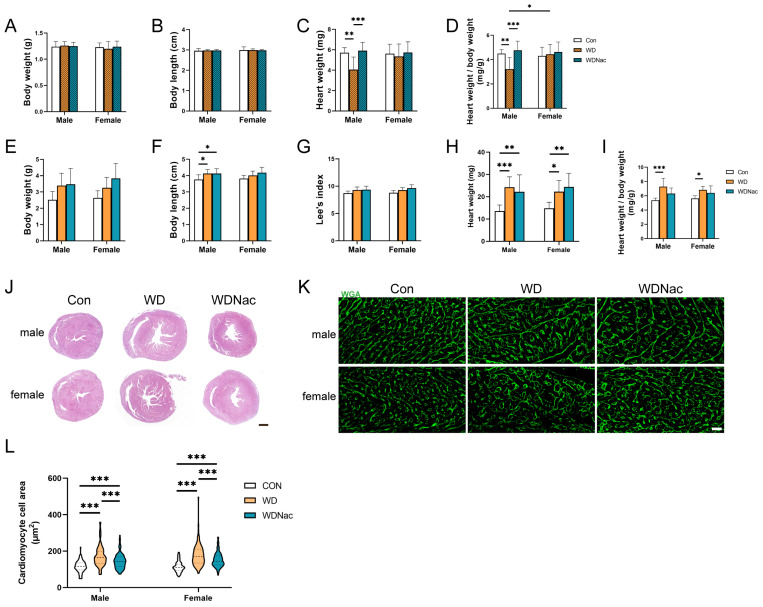
Anthropometric measurement and cardiac morphology in neonatal mice. (**A**–**D**) Body weight, body length, heart weight, and heart weight to body weight ratio in offspring on postnatal day 1 (*n* = 9–13). (**E**–**I**) Body weight, body length, Lee’s index, heart weight, and heart weight to body weight ratio in offspring on postnatal day 7 (*n* = 9–14). (**J**) Representative H&E staining of mid-cardiac sections of offspring on postnatal day 7. Bar = 500 μm. (**K**,**L**) Representative images of wheat-germ agglutinin (WGA)-stained transverse cardiac sections and quantification of cardiomyocyte cell area in offspring on postnatal day 7 (*n* = 5). Bar = 20 μm. *** *p* < 0.001, ** *p* < 0.01, * *p* < 0.05.

After birth, the physical growth, heart weight, and heart morphology of the offspring were evaluated. The absolute heart weight and relative heart weight adjusted for body weight (the ratio of heart weight to body weight) are crucial indicators for assessing cardiomyocyte hypertrophy and cardiac tissue remodeling. Our results revealed that on the first day after birth, male offspring in the WD group exhibited significantly lower absolute (*p* = 0.0054) and relative (*p* = 0.0012) heart weights than those in the control group, whereas N-acetylcysteine treatment reversed these changes (*p_absolute_* = 0.0009, *p_relative_* < 0.0001) despite no significant differences in body weight or body length among all offspring (Figure 2A–D). However, on the seventh day after birth, male offspring in the WD group had a significantly longer body length than the male offspring did in the control group (*p* = 0.0243), whereas there were no significant changes in body weight or Lee’s index. Maternal N-acetylcysteine treatment did not affect the physical development of the offspring during the neonatal period (Figure 2E–G). Notably, the absolute (*p_male_* = 0.0001, *p_female_* = 0.0384) and relative (*p_male_* < 0.0001, *p_female_* = 0.0167) heart weights of the offspring in the WD group were significantly greater than those of the offspring in the control group (Figure 2H,I).

Furthermore, we examined the cardiac morphological structure of neonatal offspring. HE staining revealed signs of left ventricular remodeling, such as enlarged ventricles, in the offspring of the WD group, while the offspring in the WDNac group did not exhibit such changes (Figure 2J). WGA staining and related statistical analysis demonstrated that the cross-sectional area of the left ventricular cardiomyocytes was significantly greater in the WD group than in the control group, while it was significantly reduced in the WDNac group (Figure 2K,L). These findings suggested that the features of left ventricular cardiomyocyte hypertrophy and remodeling induced by a maternal Western diet could be alleviated using N-acetylcysteine administration.

### 3.2. N-Acetylcysteine Alleviates Myocardial Interstitial Fibrosis and Extracellular Matrix Remodeling Induced by a Maternal Western Diet

**Figure 3:**
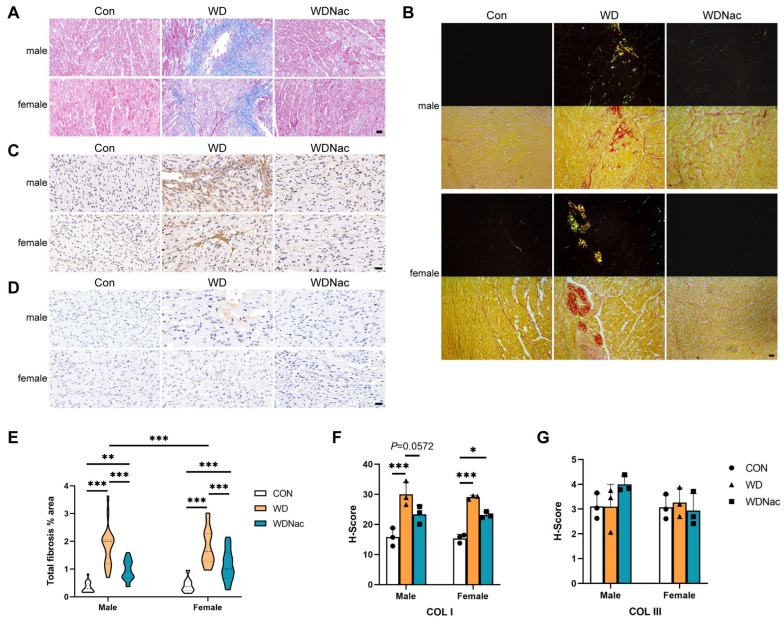
Myocardial interstitial fibrosis in offspring on postnatal day 7. (**A**) Representative images of Masson’s trichrome-stained transverse cardiac sections in neonatal offspring; areas positive for collagen were stained blue. (**B**) Representative images of ventricular mid-cardiac sections stained with picrosirius red and polarized light detection in offspring: areas positive for collagen were stained red in picrosirius red staining, areas positive for collagen type I were stained yellow, and collagen type III areas were stained green in the polarized light detection. (**C**,**D**) Representative images of collagen type I and collagen type III immunohistochemical-stained transverse cardiac sections in offspring. (**E**) Quantification of total fibrosis expressed as a percentage of tissue area by Masson’s trichrome staining (*n* = 5). (**F**,**G**) Collagen type I and collagen type III expression score (H-score) in transverse cardiac sections using immunohistochemical staining (*n* = 3). Bar = 20 μm. *** *p* < 0.001, ** *p* < 0.01, * *p* < 0.05.

The extracellular matrix plays a pivotal role in directing cell behavior in normal and pathological tissues. Masson staining and associated analysis revealed that the collagen content in the myocardial tissue of the offspring in the WD group was significantly greater than that in the myocardial tissue of the offspring in the control group, with more severe manifestations observed in male offspring than in their female counterparts. Notably, following N-acetylcysteine treatment, both male and female offspring exhibited a marked reduction in collagen content within their myocardial tissue (Figure 3A,E). Although the picrosirius red stain alone lacks selectivity in binding to the collagen network, its specificity surpasses that of other common collagen stains when used in conjunction with polarized light detection. In this study, picrosirius red staining and polarized light detection confirmed the findings observed above, revealing the primary presence of collagen type I (yellow) and collagen type III (green) in the WD group (Figure 3B).

To further identify and quantify the collagen fiber subtypes altered in the myocardium of dams fed a Western diet with or without N-acetylcysteine treatment, immunohistochemical staining for collagen type I and type III was performed. The results indicated that the elevated collagen content in the myocardial tissue of the offspring in the WD group, primarily attributed to the increase in collagen type I, was significantly lower in male offspring in the WDNac group (Figure 3C,D,F,G). These findings collectively confirmed that the myocardial interstitial fibrosis induced by a maternal Western-style diet could be prevented by N-acetylcysteine supplementation during pregnancy.

### 3.3. N-Acetylcysteine Attenuates Cardiomyocyte Apoptosis and Dysfunction Resulting from a Maternal Western Diet

**Figure 4:**
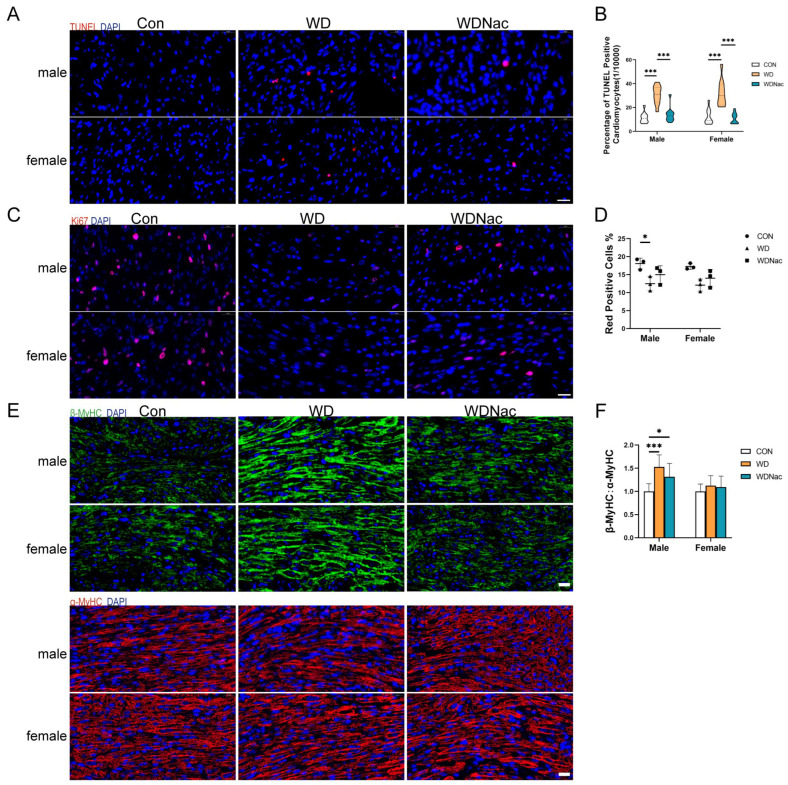
Apoptosis, proliferation and function of cardiomyocytes in offspring on postnatal day 7. (**A**,**B**) Representative images of TUNEL immunofluorescent-stained transverse cardiac sections and quantification of TUNEL-positive cardiomyocytes in offspring (*n* = 5). Bar = 20 μm. (**C**,**D**) Representative images of Ki67 immunofluorescent-stained transverse cardiac sections and quantification of Ki67-positive cardiomyocytes in offspring (*n* = 3). Bar = 20 μm. (**E**,**F**) Representative images of α-MyHC and β-MyHC immunofluorescent staining and quantification of MyHC:α-MyHC ratio in transverse cardiac sections of neonatal offspring (*n* = 5). Bar = 20 μm. *** *p* < 0.001, * *p* < 0.05.

The proliferation and apoptosis of cardiomyocytes are crucial functions for the overall contractile pumping function of the heart, making them vital to individual health. The status of cell apoptosis and proliferation in the myocardial tissue of the offspring across different groups was investigated in this study. TUNEL and Ki-67 immunofluorescence staining demonstrated a significant increase in the proportion of apoptotic cells and a decrease in the proportion of proliferating cells in the myocardial tissue of the offspring in the WD group, with more pronounced effects observed in males than in females. However, the administration of N-acetylcysteine to the dams during pregnancy significantly attenuated apoptosis induced by the maternal Western diet (Figure 4A–D).

Myosin, specifically the myosin heavy chain (MyHC), plays a pivotal role in the structure and function of cardiomyocytes. Notably, the MyHC proteins encoded by the MYH6 and MYH7 genes, namely, the α-MyHC and β-MyHC proteins, respectively, are crucial for maintaining myocardial function. Changes in the expression levels and ratios of these proteins can indicate alterations in myocardial hypertrophy and function. Immunohistochemical staining for α-MyHC and β-MyHC in mouse myocardial tissue revealed that compared to those in the control (Con) group, male offspring in the WD group exhibited significantly increased concentrations of β-MyHC and a marked increase in the β-MyHC:α-MyHC ratio, primarily due to an increase in the β-MyHC concentration. Notably, this imbalance, characterized by increased β-MyHC concentration, was significantly ameliorated in male offspring treated with N-acetylcysteine during pregnancy (WDNac group). However, in female offspring, there were no significant differences in the α-MyHC or β-MyHC concentration or the β-MyHC:α-MyHC ratio among the three groups (Figure 4E,F). These results suggested that prenatal N-acetylcysteine treatment effectively alleviates the myocardial contractile dysfunction of male offspring induced by a maternal Western diet.

To accurately evaluate the cardiac functional changes in the offspring, Doppler echocardiography was conducted to assess crucial ventricular remodeling parameters, such as left ventricular internal diameter, wall and septal thicknesses, and left ventricular mass during both the systolic and diastolic phases in the neonatal mice among the three groups. Compared to those in the Con group, the offspring in the WD group exhibited a significantly thicker interventricular septum during the systolic phase (IVS;s) and diastolic phase (IVS;d). Additionally, the left ventricular mass (LV mass) was significantly greater in male offspring in the WD group than in the male offspring in the control group, although the left ventricular ejection fraction (EF) and fractional shortening (FS) did not significantly differ between the two groups. Notably, prenatal N-acetylcysteine treatment partially improved and corrected the structural changes in the left ventricle of male offspring from WD dams (WDNac), particularly the reduction in IVS thickness, which approached that of the control group. However, in female offspring, no significant differences were observed in left ventricular structural parameters when comparing the WDNac group to the WD group, indicating a clear sex difference (Table 1). These findings suggest that, as expected, prenatal N-acetylcysteine supplementation effectively alleviates the left ventricular remodeling of male offspring of dams fed a maternal Western diet.

### 3.4. N-Acetylcysteine Recovers Disrupted Oxidative Phosphorylation in Offspring from Dams Fed a Western Diet

**Figure 5:**
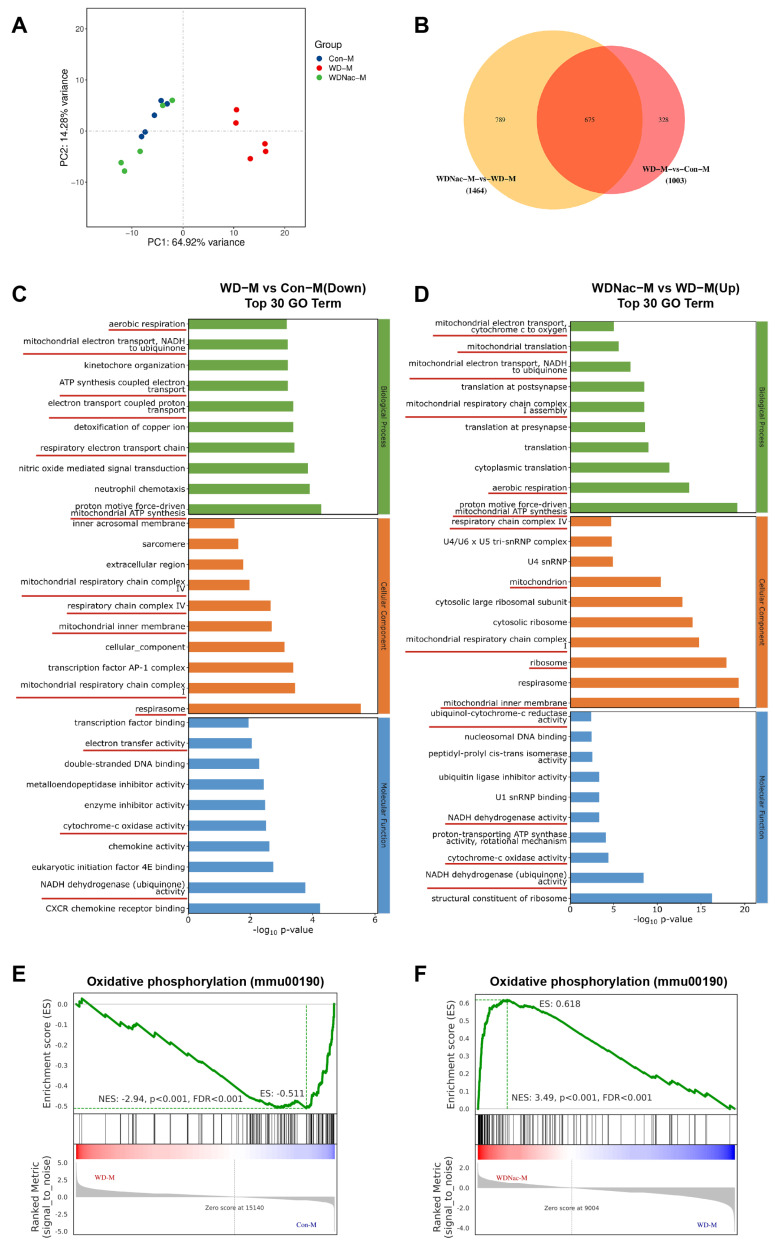
Identification and functional analysis of differentially expressed genes (DEGs) in the hearts of male offspring on postnatal day 7 (*n* = 5). (**A**) The PCA plot shows the ideal discrimination of controls (Con-M), maternal Western diet-induced cardiomyopathy (WD-M), and maternal Western diet-induced cardiomyopathy with N-acetylcysteine treatment (WDNac-M). (**B**) The Venn diagram illustrates the number of DEGs and their overlapping genes. (**C**,**D**) Functional enrichment analysis using Gene Ontology (GO) databases reveals the top 30 terms enriched on DEGs, with green indicating biological process, orange indicating cellular component, and blue indicating molecular function, adjusted *p* < 0.05. The terms related to mitochondrial function are underlined. (**E**,**F**) Gene set enrichment analysis (GSEA) was performed to identify distinct phenotypic differences between the different groups.

**Figure 6:**
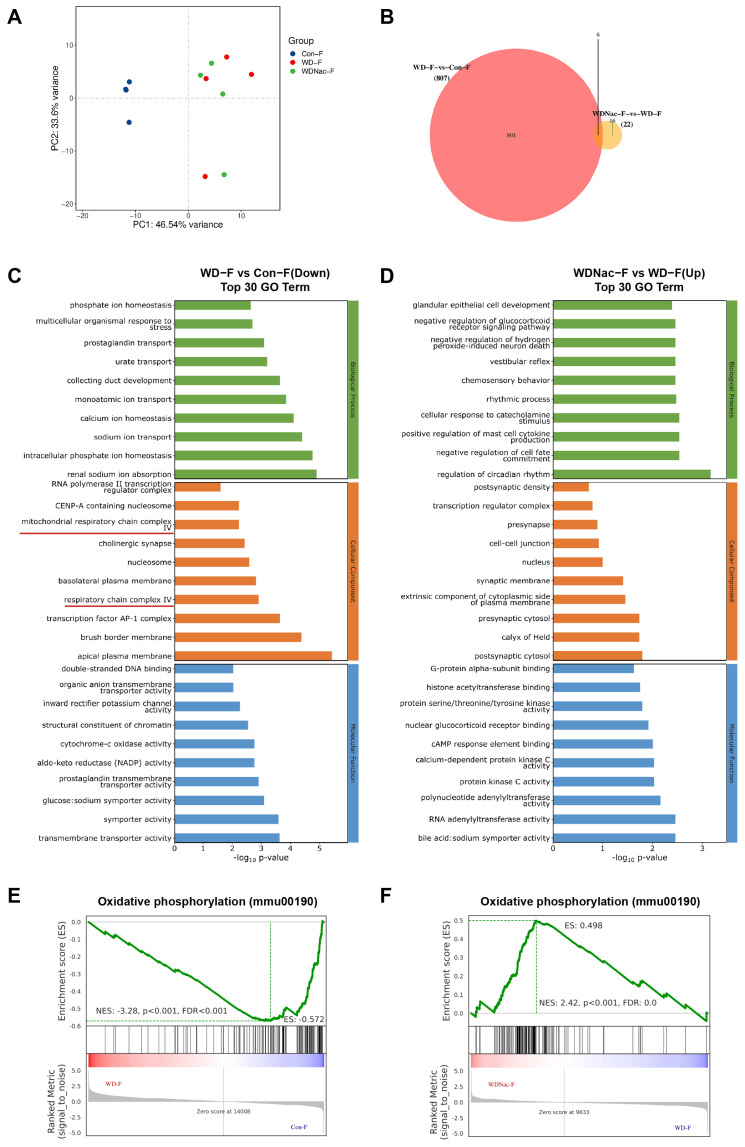
Identification and functional analysis of differentially expressed genes (DEGs) in the hearts of female offspring on postnatal day 7 (*n* = 4). (**A**) The PCA plot shows the ideal discrimination of controls (Con-F), maternal Western diet-induced cardiomyopathy (WD-F), and maternal Western diet-induced cardiomyopathy with N-acetylcysteine treatment (WDNac-F). (**B**) The Venn diagram illustrated the number of DEGs and their overlapping genes. (**C**,**D**) Functional enrichment analysis using Gene Ontology (GO) databases reveals the top 30 terms enriched on DEGs, with green indicating biological process, orange indicating cellular component, and blue indicating molecular function, adjusted *p* < 0.05. The terms related to mitochondrial function are underlined. (**E**,**F**) Gene set enrichment analysis (GSEA) was performed to identify distinct phenotypic differences between different groups.

Maternal exposure to a Western diet may permanently alter the genetic information of offspring through epigenetic modification, which may be prevented by intervention. Therefore, we explored the underlying molecular mechanisms by which N-acetylcysteine relieves cardiomyopathy induced by a maternal Western diet. The RNA sequencing analysis of the heart under maternal diet conditions revealed distinct transcriptome patterns among the three groups. Notably, a maternal Western diet resulted in sexual dimorphism in the hearts of offspring. In male offspring, principal component analysis (PCA) suggested that fifteen samples could be divided into three major groups, which revealed good repeatability within five samples of each group (Figure 5A). Differentially expressed genes (DEGs) were defined as those with q values < 0.05 and |log2(foldchange)| > 1. There were 1003 genes (718 upregulated and 285 downregulated) differentially expressed in the hearts of male offspring from dams fed a Western diet compared with those from dams fed a control diet, and 1464 genes (564 upregulated and 900 downregulated) were differentially expressed in the hearts of male offspring from dams fed a Western diet and treated with N-acetylcysteine compared with those from dams only fed a Western diet. In addition, there were 675 genes that overlapped between the cluster of WD-M vs. Con-M and that of WDNac-M vs. WD-M (Figure 5B). Gene Ontology (GO) analysis revealed a significant enrichment of DEGs in male mice, including downregulated enriched pathways in the WD-M vs. Con-M groups and upregulated enriched pathways in the WDNac-M vs. WD-M groups. These enriched pathways mainly included aerobic respiration, mitochondrial electron transport-NADH to ubiquinone, mitochondrial respiratory chain complex I, mitochondrial respiratory chain complex IV, respirasome, and NADH dehydrogenase (ubiquinone) activity (Figure 5C,D). In contrast, 807 DEGs (647 upregulated and 160 downregulated) were detected in the WD-F group compared with the Con-F group, and 22 DEGs (13 upregulated and 9 downregulated) were detected in the WDNac-F group compared with the WD-F group, with a minimum of 6 genes overlapping between them (Figure 6A,B). GO analysis revealed the significant enrichment of downregulated genes in the WD-F vs. Con-F groups and upregulated genes in the WDNac-F vs. WD-F groups, with the DEGs mainly related to the mitochondrial respiratory chain (Figure 6C,D). These disparities in DEG numbers and the limited overlap suggested the varying responses of male and female offspring to a maternal Western diet and the specific effectiveness of N-acetylcysteine in preventing myocardial damage in male offspring from dams fed a Western diet.

Gene set enrichment analysis (GSEA) revealed that the maternal Western diet was associated with multiple signaling pathways. The oxidative phosphorylation pathway was screened out due to its relationship with mitochondrial function, and most DEGs were involved in this pathway. Compared to the control group (Con), the gene set linked to the oxidative phosphorylation pathway is downregulated in the WD group but exhibits upregulation in the WDNac group, although the degree of enrichment differed between males and females (Figure 5E,F and Figure 6E,F). These results suggested that the antioxidant N-acetylcysteine may mitigate the maternal Western diet-induced cardiomyopathy through oxidative stress and oxidative phosphorylation signaling pathways.

### 3.5. Boosting GSH Levels and GPx Activity with N-Acetylcysteine Effectively Scavenged Mitohondrial ROS in Male Offspring of Western Diet-Fed Dams

**Figure 7:**
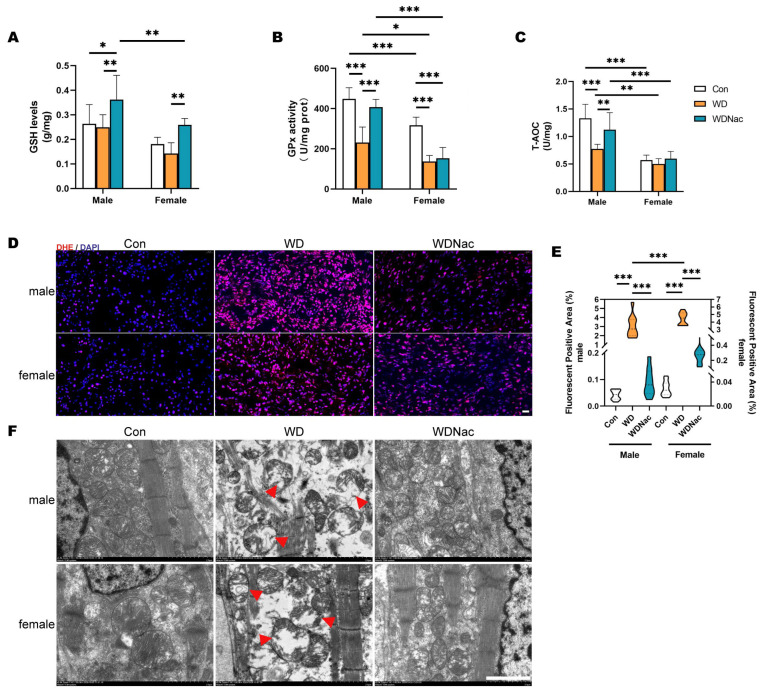
Antioxidant defense system in myocardium of neonatal offspring on postnatal day 7. (**A**–**C**) Glutathione (GSH) concentrations, glutathione peroxidase (GPx) activity, and the total antioxidant capacity (T-AOC) in the myocardial tissue of offspring (*n* = 6–14). (**D**,**E**) Representative images of fluorescent probe dihydroethidium (DHE) staining and the quantification of the DHE-positive area in the myocardial tissue of offspring (*n* = 5). Bar = 20 μm. (**F**) Mitochondrial morphology the in myocardium of offspring shown via transmission electron microscopy; red triangles indicate abnormal mitochondria. Bar = 2 μm. *** *p* < 0.001, ** *p* < 0.01, * *p* < 0.05.

To confirm the influence of N-acetylcysteine on oxidative phosphorylation, we evaluated the antioxidant defense system, including the crucial antioxidant enzyme glutathione peroxidase (GPx) and the antioxidant glutathione (GSH). GSH plays an important role in the antioxidant defense system as a natural antioxidant, and N-acetylcysteine treatment significantly increased GSH concentrations in the hearts of offspring mice, with sex-specific differences observed (Figure 7A). Additionally, the results showed that the maternal Western diet significantly reduced GPx activity in the myocardial tissue of offspring and that N-acetylcysteine treatment effectively increased GPx activity in the hearts of male offspring but not in those of female offspring (Figure 7B). Furthermore, N-acetylcysteine treatment significantly restored the decrease in total antioxidant capacity (T-AOC) resulting from the maternal Western diet in the myocardial tissue of male offspring (Figure 7C). These findings suggested that N-acetylcysteine treatment during gestation effectively mitigated the impact of the maternal Western diet on the antioxidant defense system and GSH redox system in the myocardial tissue of offspring, with sex-specific differences evident.

To validate the scavenging effect of GSH on reactive oxygen species (ROS), we employed fluorescence spectroscopy using the fluorescent probe dihydroethidium (DHE) to investigate the ROS levels in the hearts of the offspring. The results demonstrated that the intensity of red fluorescence and the proportion of fluorescently positive areas of DHE in myocardial tissue were significantly greater in the WD group than in the control group, with a more pronounced increase observed in males than in females. The administration of N-acetylcysteine significantly reduced the DHE fluorescence intensity and area in the heart tissue of the offspring of the WD group (Figure 7D,E). This finding suggested that the maternal Western diet significantly elevated ROS levels in the hearts of offspring and that N-acetylcysteine treatment during gestation substantially mitigated ROS production in the hearts of neonatal offspring.

To confirm the reparative effect of N-acetylcysteine on mitochondrial damage induced by the maternal Western-style diet, we examined the ultrastructure of mitochondria in the cardiac tissue of the offspring using transmission electron microscopy. The results revealed that compared to those in the Con group, the offspring in the WD group exhibited a decrease in mitochondrial number and a disrupted arrangement within their cardiac muscle fibers. Importantly, in the WD group, the mitochondria were swollen and damaged, with shortened, reduced, fractured, and displaced cristae, as well as a dissolved matrix and vacuole formation. However, following treatment with N-acetylcysteine, the WDNac group exhibited a significant increase in the number of mitochondria in cardiac muscle fibers, with a normal morphology, orderly arrangement, and clearly visible cristae. These abnormalities in mitochondrial morphology and volume were notably alleviated in the WDNac group (Figure 7F). These findings suggest that N-acetylcysteine effectively restores mitochondrial morphological damage in the hearts of neonatal offspring, the latter resulting from the consumption of a Western-style diet by dams.

**Figure 8:**
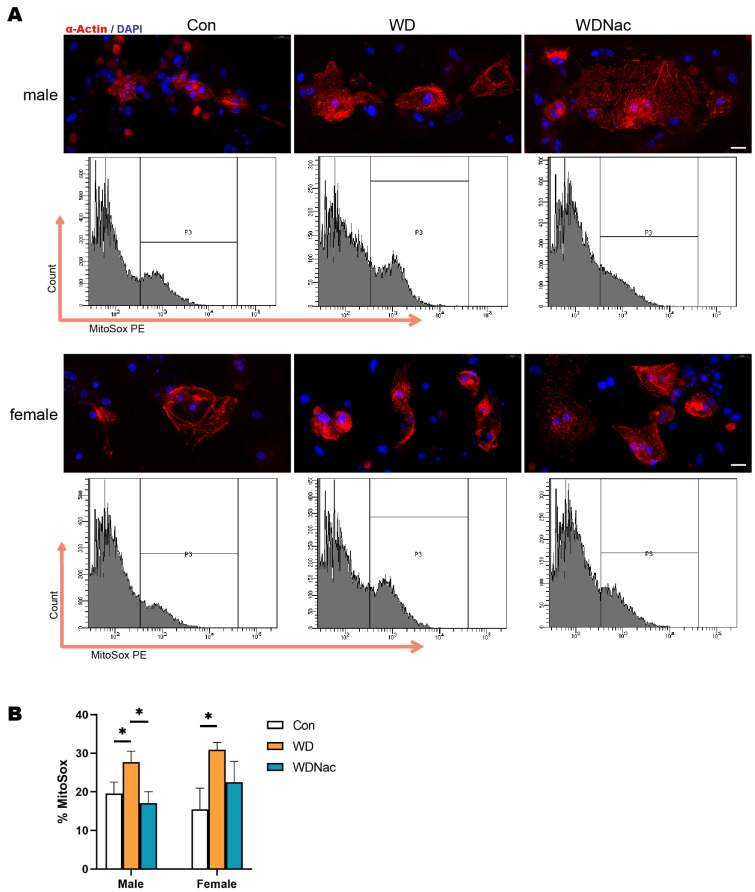
Mitochondrial ROS in primary cardiomyocytes of neonatal offspring on postnatal day 7. (**A**,**B**) Level of mitochondrial ROS-specific fluorescent probe MitoSOX in primary cardiomyocytes (identified using a-Actin antibodies) of offspring shown via flow cytometry (*n* = 3). Bar = 20 μm. * *p* < 0.05.

Typically, mitochondria serve as the primary source of ROS, which are primarily generated through nicotinamide adenine dinucleotide phosphate (NADPH) oxidases (NOX) and mitochondrial respiratory chain complexes. Therefore, we used the mitochondrial ROS-specific fluorescent dye MitoSOX to investigate changes in mitochondrial ROS (mtROS) in primary cardiomyocytes, which were identified using a-Actin antibodies, from neonatal offspring from different groups. Our results demonstrated that the percentage of mtROS in cardiomyocytes from both male and female offspring in the WD group was significantly greater than that in cardiomyocytes from the Con group. However, in the WDNac group, the mtROS levels in the cardiomyocytes of the male offspring were significantly reduced (Figure 8A,B). The alteration in mtROS levels aligns with the changes observed in myocardial ROS levels, indicating that N-acetylcysteine treatment during pregnancy can effectively attenuate the overproduction of mtROS, which may be attributed to mitochondrial dysfunction in the hearts of neonatal offspring as a result of a maternal Western diet.

## 4. Discussion

The findings from our study suggested that the accumulation of ROS, triggered by a maternal Western diet, potentially contributes to the perturbation of cardiomyocyte metabolism and function, ultimately precipitating adverse myocardial remodeling and interstitial fibrosis in neonatal offspring. Moreover, our data suggest that these effects may be mediated by the oxidative phosphorylation pathway. Notably, augmenting GSH synthesis through N-acetylcysteine administration protected against cardiomyopathy induced by a maternal Western diet, as evidenced by elevated GSH levels, enhanced GPx activity, and the effective scavenging of mitochondrial ROS.

Excitingly, we found that the protective effect of N-acetylcysteine on maternal Western diet-induced cardiomyopathy in the offspring was dependent on sex, with male offspring exhibiting cardiac protection after N-acetylcysteine supplementation through the regulation of the GSH system and redox homeostasis; similar effects were not observed in females. Indeed, the sex differences in fetal-originated diseases, particularly those linked to cardiomyopathy induced by prenatal glucolipotoxicity, are profound and intricate [5]. The mechanisms involved in sex differences in the developmental origins of cardiometabolic disease involve intricate interplay among sex-specific gene expression, steroid hormones, metabolic hormones, and epigenetic modifications during fetal development [25,26]. These observations underscore the need for sex-specific research to identify differences in the developmental origins of health and disease. Additionally, a thorough comprehension of these sex-specific differences is vital for developing targeted interventions that can effectively protect the cardiovascular health of both male and female offspring.

Heart weight serves as a crucial indicator for assessing cardiomyopathy. Our findings revealed that the offspring in the maternal Western diet group exhibited a lower heart weight than did those in the control group on the first day of life but had a greater heart weight on the seventh day. These paradoxical results can be explained by the compromised proliferative capacity of cardiomyocytes, a key feature of fetal heart development [27], in offspring exposed to a maternal Western diet. This impairment led to a decrease in heart weight on the first day of life. The accumulation of ROS triggers the arrest of cardiomyocyte proliferation shortly after birth, whereas effectively scavenging ROS can significantly prolong the regenerative potential of these cells [28]. Once mammals are born, cardiomyocytes transition from a hypoxic environment to one with high oxygen levels, resulting in cell cycle arrest and the loss of proliferative capacity [29]. In mice, the window of cardiac regenerative capacity is limited to only seven days under normal conditions, during which the significance of cardiomyocyte proliferation diminishes during heart development and maturation. The increased collagen production and deposition triggered by the maternal Western diet ultimately led to an increase in heart weight in the WD group on the seventh day of life. Ultimately, the administration of N-acetylcysteine normalized aberrant processes at various stages, returning them to their normal patterns in this study.

In this study, we employed N-acetylcysteine treatment, given its status as a GSH precursor and its direct and indirect antioxidant activities. Interestingly, in terms of its impact on markers of oxidative stress, the effects of N-acetylcysteine, which serve as a GSH donor, exceed those of GSH itself [30]. This is primarily attributed to the limited absorption of orally ingested GSH, stemming from the action of intestinal enzymes, particularly gamma-glutamyl transpeptidase (GGT), which degrades GSH [31]. GSH, composed of three amino acids—glycine, cysteine, and glutamic acid—serves as an efficient scavenger of ROS. Notably, cysteine is the primary limiting factor in GSH synthesis [32]. Numerous studies have demonstrated that N-acetylcysteine is efficiently absorbed by the intestine and that N-acetylcysteine supplementation effectively elevates GSH levels and significantly increases total antioxidant status in humans and animals [33,34,35], observations that are consistent with the findings of our investigation.

Owing to its abundant mitochondria and high energy demand, the heart is at high risk of oxidative damage, making it crucial to maintain redox homeostasis and efficiently scavenge ROS to protect against oxidative stress and associated pathologies. We and other researchers have reported that the accumulation of ROS disrupts the normal physiology of cardiomyocytes by modulating the expression of genes involved in mitochondrial biogenesis, damage, and antioxidant defense mechanisms following oxidative stress [7,36,37]. Epigenetic alterations may be linked to concurrent changes in gene regulation, potentially contributing to the pathophysiology of cardiac dysfunction [38]. As expected, the beneficial effects of N-acetylcysteine on reducing ROS levels in the hearts of offspring from WD-fed dams were demonstrated by the increase in glutathione levels and GPx activity, thus protecting mitochondria and their function. Consistent with our results, N-acetylcysteine treatment in type 2 diabetic mice has been shown to effectively preserve the expression of antioxidant enzymes, attenuate ROS production in diabetic ischemic limbs, and reverse the levels of phosphorylated IRS-1, Akt, eNOS, and plasma TNF-α [39]. N-acetylcysteine has also been shown in preclinical studies to have the potential to improve many diabetes- and obesity-associated complications, including the inhibition of oxidative stress-induced heart damage, as highlighted in recent reviews [40,41].

Myocardial interstitial fibrosis is a process caused by multiple factors, including the activation of cardiac fibroblasts, cardiomyocyte apoptosis, and endothelial cell injury. Moderate extracellular matrix protein deposition sustains the integrity of the structure and function of the heart through compensation, but extensive fibrosis can impair heart function. ROS diffuse throughout the cytoplasm, triggering the activation of redox-sensitive protein kinases. These kinases play a pivotal role in promoting fibrosis by stimulating fibroblast proliferation, cardiomyocyte apoptosis, and endothelial cell injury. The results of our study demonstrated that antioxidant treatment profoundly mitigated the increase in cardiac collagen content, as well as ROS, induced by the maternal diet. ROS play a key role in modulating the extracellular matrix by enhancing the protein expression of TGF-β1, alpha smooth muscle actin, collagen I, and collagen III, leading to the activation of cardiac fibroblasts [42]. A significant increase in myocardial fibroblast activation and interstitial collagen deposition can result in the development of restrictive diastolic dysfunction with increased filling pressure [43]. As such, the scavenging of ROS effectively modulates the inflammatory microenvironment and inhibits the proliferation of cardiac fibroblasts by causing the constitutive expression of HIF-1α, thereby suppressing fibrosis and promoting heart repair following myocardial infarction [44]. Concurrently, ROS can also trigger apoptosis, and damaged tissue elicits an inflammatory response, releasing proinflammatory cytokines and chemokines. Fibroblasts undergo differentiation into myofibroblasts and produce collagen in response to the transduction of proinflammatory signals, such as transforming growth factor-β (TGF-β)/SMADs and Wingless (Wnt)/β-catenin [45,46]. Therefore, it can be inferred that ROS clearance could effectively alleviate pathological processes such as apoptosis and fibrosis in the myocardium, consistent with our findings in this study.

Oxidative stress plays a pivotal role in initiating epigenetic alterations, ultimately leading to the pathogenesis of cardiomyopathy. DNA methylation within cardiac fibroblasts results in the deactivation of the tumor suppressor gene RASSF1A, simultaneously activating ERK1/2, thereby promoting fibroblast proliferation and ultimately leading to the development of cardiac fibrosis. Extracellular superoxide dismutase (EC-SOD) has a remarkable ability to attenuate RASSF1A gene methylation and alleviate cardiac fibrosis induced by hypoxia [47]. Alterations in the redox state of conserved cysteine residues within class II histone deacetylases (HDACs), a class of epigenetic regulatory enzymes, profoundly modulate both their nucleocytoplasmic shuttling and the activity of critical genes linked to cardiomyocyte hypertrophy [48]. An inhibitor of HDACs was shown to mitigate cardiomyocyte apoptosis, decrease the generation of ROS, and promote angiogenesis in a mouse model of type II diabetes induced by a high-fat diet [49]. Moreover, an HDAC inhibitor enhanced glucose metabolism and glycolysis in cardiomyocytes, increased cellular oxidative phosphorylation, and improved the function of myocardial mitochondria in diabetic cardiomyopathy [50]. These studies indicated that suppressing oxidative stress and ROS could significantly alleviate myocardial mitochondrial damage and oxidative phosphorylation triggered by nutritional insults. Consistent with our findings, transmission electron microscopy revealed that N-acetylcysteine treatment alleviated morphological disruptions in myocardial mitochondria and reduced mtROS levels in the heart of the WD group, suggesting that N-acetylcysteine may exert a protective effect on the cardiomyopathy observed in male offspring from dams fed a Western diet, likely through the modulation of the oxidative phosphorylation pathway, as evidenced using RNA sequencing. Recent research has suggested that oxidative stress can impact cardiac fibrosis by modulating epigenetic mechanisms and that epigenetic modifications may, in turn, mitigate cardiac fibrosis by reducing oxidative stress [38]. Multiple antioxidant defense systems have evolved to prevent the hazardous accumulation of ROS. Among these, the glutathione/glutathione-peroxidase and thioredoxin/thioredoxin-reductase systems stand out as the most potent endogenous antioxidant mechanisms. In cardiovascular diseases related to oxidative stress, reduced thioredoxin (Trx) scavenges ROS and exerts a protective effect to maintain cellular redox balance [51]. Thioredoxin reductase (TrxR) plays a pivotal role in sustaining GSH homeostasis within cells by efficiently catalyzing the reduction in GSSG back to GSH. Intriguingly, recent findings have revealed a remarkable interplay between the thioredoxin and glutathione systems, where they not only provided electrons crosswise but also functioned as backup systems for each other [52,53]. Further research may prioritize delving into the role of the Trx/TrxR systems in modulating ROS levels and elucidating the vital crosstalk between the thioredoxin and glutathione antioxidant pathways in oxidative stress-related cardiac damage. Given DHE probes’ limitations in quantifying ROS in fixed heart sections, adopting more suitable methods to accurately assess ROS changes in cardiac tissues is crucial.

To gain a profound understanding of the specific impacts of a Western diet on offspring health, it is essential to investigate and compare the effects of various dietary interventions during pregnancy in further studies. Conducting longitudinal studies is vital to comprehensively assess the long-term benefits and potential adverse effects of N-acetylcysteine treatment. These studies must be performed in larger, more varied animal models to ensure the findings are comprehensive and representative of a broad range of biological conditions. Ensuring the safety and efficacy of N-acetylcysteine for pregnant women necessitates the design and initiation of well-planned clinical trials that thoroughly scrutinize its safety profile and therapeutic benefits. Furthermore, a longitudinal evaluation of the cardiovascular health of the offspring is crucial to detect any potential long-term consequences resulting from prenatal exposure to N-acetylcysteine. This comprehensive approach will provide vital insights into the appropriateness of its use in this vulnerable population and its potential implications on the cardiovascular health of future generations.

## 5. Conclusions

N-acetylcysteine mitigated cardiomyopathy induced by a maternal Western diet by bolstering glutathione synthesis and enhancing GPx activity, thereby scavenging mitochondrial ROS and modulating oxidative phosphorylation pathways. These findings suggest that GSH synthesis presents itself as a promising avenue for therapeutic intervention in cardiomyopathy caused by maternal glycolipid metabolic disorders.

## Figures and Tables

**Table 1:** Cardiac morphology and systolic function in neonatal mice.

	Con	WD	WDNac	*p* Value
Male	*n* = 9	*n* = 10	*n* = 9	
LVPW;s	0.81 ± 0.08	0.92 ± 0.13	0.85 ± 0.22	0.2977
LVPW;d	0.48 ± 0.05	0.61 ± 0.09	0.55 ± 0.17	0.1425
LVID;s	0.85 ± 0.16	1.02 ± 0.17	0.94 ± 0.21	0.1539
LVID;d	1.73 ± 0.21	2.01 ± 0.26	1.86 ± 0.30	0.1062
IVS;s	0.80 ± 0.08	1.01 ± 0.12 ^a,b^	0.77 ± 0.13	0.0006
IVS;d	0.52 ± 0.08	0.66 ± 0.10 ^a,b^	0.47 ± 0.08	0.0008
LV Mass	13.07 ± 2.92	23.67 ± 8.45 ^a,b^	14.83 ± 6.69	0.0093
LVEF	65.02 ± 4.36	63.01 ± 3.07	63.39 ± 3.40	0.4901
LVFS	31.44 ± 5.35	29.27 ± 3.53	31.80 ± 8.31	0.6556
Female	*n* = 7	*n* = 8	*n* = 10	
LVPW;s	0.78 ± 0.12	0.98 ± 0.28	0.98 ± 0.20	0.1234
LVPW;d	0.49 ± 0.08	0.66 ± 0.27	0.62 ± 0.16	0.1974
LVID;s	0.85 ± 0.18	0.89 ± 0.17	0.93 ± 0.14	0.6165
LVID;d	1.71 ± 0.22	1.85 ± 0.23	1.92 ± 0.27	0.2376
IVS;s	0.77 ± 0.19	1.03 ± 0.22 ^a^	1.00 ± 0.17	0.0330
IVS;d	0.48 ± 0.08	0.67 ± 0.18 ^a^	0.61 ± 0.12	0.0310
LV Mass	11.99 ± 2.83	23.22 ± 12.70	21.10 ± 7.53	0.0498
LVEF	64.54 ± 4.28	64.68 ± 7.65	64.32 ± 6.38	0.9926
LVFS	30.79 ± 5.07	31.80 ± 8.31	31.18 ± 7.13	0.9613

Mean ± SD echocardiographic measurements of left ventricular morphology and systolic function in the neonatal offspring of control (Con), Western diet-induced mice (WD), and Western diet-induced mice treated with N-acetylcysteine (WDNac). A one-way ANOVA followed by a Bonferroni post hoc test was performed for statistical analysis. ^a^ *p* < 0.05 versus Con; ^b^ *p* < 0.05 versus WDNac. LVPW;s, left ventricular posterior wall thickness at end-systole. LVPW;d, left ventricular posterior wall thickness at end-diastole. LVID;s, left ventricular internal dimension at end-systole. LVID;d, left ventricular internal dimension at end-diastole. IVS;s, interventricular septum at end-systole. IVS;d, interventricular septum at end-diastole. LVEF, left ventricular ejection fraction. LVFS, left ventricular fraction shortening.

## Data Availability

Data is contained within the article.
